# The Mitochondrial Permeability Transition in Mitochondrial Disorders

**DOI:** 10.1155/2019/3403075

**Published:** 2019-05-05

**Authors:** Justina Šileikytė, Michael Forte

**Affiliations:** Vollum Institute, Oregon Health & Science University, Portland, OR 97239, USA

## Abstract

Mitochondrial permeability transition pore (PTP), a (patho)physiological phenomenon discovered over 40 years ago, is still not completely understood. PTP activation results in a formation of a nonspecific channel within the inner mitochondrial membrane with an exclusion size of 1.5 kDa. PTP openings can be transient and are thought to serve a physiological role to allow quick Ca^2+^ release and/or metabolite exchange between mitochondrial matrix and cytosol or long-lasting openings that are associated with pathological conditions. While matrix Ca^2+^ and oxidative stress are crucial in its activation, the consequence of prolonged PTP opening is dissipation of the inner mitochondrial membrane potential, cessation of ATP synthesis, bioenergetic crisis, and cell death—a primary characteristic of mitochondrial disorders. PTP involvement in mitochondrial and cellular demise in a variety of disease paradigms has been long appreciated, yet the exact molecular entity of the PTP and the development of potent and specific PTP inhibitors remain areas of active investigation. In this review, we will (i) summarize recent advances made in elucidating the molecular nature of the PTP focusing on evidence pointing to mitochondrial F_o_F_1_-ATP synthase, (ii) summarize studies aimed at discovering novel PTP inhibitors, and (iii) review data supporting compromised PTP activity in specific mitochondrial diseases.

## 1. Introduction

Situated in the cytoplasm of eukaryotic cells, mitochondria are essential for normal cell function. Notably, these dynamic, double membrane structures gained considerable attention in recent years due to their role in Ca^2+^ homeostasis, interorganelle communication, cell proliferation, and senescence, as well as the orchestration of various signaling pathways some of which determine cell commitment to death or survival [1]. Most importantly, their vital function in cell physiology is by providing the cell with energy in the form of ATP through oxidative phosphorylation (OXPHOS). The latter, taking place in the inner mitochondrial membrane (IMM), is composed of respiratory chain complexes I–IV and F_o_F_1_-ATP synthase (ATP synthase). The OXPHOS allows for ~30 molecules of ATP to be made per one molecule of glucose or 15 times more than by glycolysis.

Mitochondria also contain their own genome which encodes proteins essential for OXPHOS function. Maternally transmitted human mitochondrial DNA (mtDNA) is circular, double-stranded helix which encodes 22 transfer RNAs, 2 ribosomal RNAs, and 13 core proteins that assemble in and determine the efficiency of all but succinate dehydrogenase (complex II) complexes of respiratory chain. Its copy number varies between cell type and developmental stage and lies between 10^3^ and 10^4^ per cell to meet the energy requirements of any specific cell type at a given time. In healthy humans, mtDNA population was initially thought to be uniform or homoplasmic, although recent studies suggest that this is only true for ~10% of individuals [2]. Upon cell division, mtDNA replicates and mitochondria are randomly segregated between daughter cells. Consequently, mutations in the mitochondrial genome give rise to heteroplasmy where normal and mutant mtDNA populations coexist resulting in genetic drift toward either pure mutant or wild type [3]. Over time, the percentage of mutant alleles may increase leading to decline in bioenergetic capacity. Once the threshold is reached, mitochondria fail to make enough energy and symptoms appear. Over 200 [4] debilitating, life-threatening, and therapeutically challenging diseases, termed mitochondrial diseases, have been linked to mutations in both mtDNA and nuclear DNA encoding for mitochondrially localized proteins. Major difficulties, first diagnosing the disease and then providing a treatment, lie in the complexity and heterogeneity of these disorders both in terms of genetic variation and clinical phenotypes. Yet, they all share a common element—decreased energy supply as a consequence of mitochondrial dysfunction. Within this group of disorders, commonly observed mitochondrial abnormalities include mitochondrial network fragmentation [5, 6], decreased OXPHOS capacity [7], increased reactive oxygen species (ROS) [8–10], and Ca^2+^ deregulation and alterations in mitochondrial ultrastructure [11–15]. All of these features are consistent with impaired regulation of the mitochondrial permeability transition pore (PTP), a conserved physiological process in mitochondria of all eukaryotes.

## 2. The Enigma of the Mitochondrial Permeability Transition

The PTP is a cyclosporine A- (CsA-) sensitive high-conductance channel in the IMM which is triggered by Ca^2+^ and potentiated by ROS. Once activated, it allows for unselective diffusion of <1500 Da solutes and water across the IMM. Two states of channel openings have been identified: short in duration, so-called flickering, and long-lasting openings. The former are thought to serve a physiological role by allowing for a quick exchange of solutes (e.g., Ca^2+^, oxygen radicals) between the mitochondrial matrix and the cytosol required for signaling [16]. Long-lasting openings result in mitochondrial depolarization, ATP consumption rather than generation in attempt to maintain IMM potential [17], burst in ROS, impaired cellular Ca^2+^ homeostasis, mitochondrial swelling, and release of proapoptotic factors into the cytosol to initiate cell death [18]. Thus, the openings of long duration are detrimental to mitochondria and mark the point of no return in cell life and death.

While the great deal of information has been collected about the regulation of the PTP (see [18–21]), its exact structural components remain the province of further experimentation. The initial belief that the PTP forms at the adjoining sites of the IMM and outer mitochondrial membrane (OMM) through association of a variety of proteins (e.g., VDAC, TSPO, and adenine nucleotide transporter (ANT) [22–24] among others) in each membrane has not been supported by rigorous genetic tests [25–28]; mitochondria missing these proteins still displayed a CsA-sensitive PTP opening (see [18] for extensive review). These findings prompted a multidisciplinary resurgence in the quest for the identification of the proteins that form the regulated channel defined as the PTP.

### 2.1. ATP Synthase as a Structural Component of the PTP

It is important to recognize that the PTP is exclusively an IMM event [23, 29]. Consequently, recent studies suggest that ATP synthase forms the long-sought PTP [30–35], but exact molecular mechanism of pore formation within the IMM or ATP synthase specifically has yet to be established. Mitochondrial ATP synthase is evolutionary conserved enzyme of the IMM, whose main function is to make ATP from ADP and phosphate. This multisubunit complex is made of 17 different proteins that are assembled into two main domains, the membrane extrinsic catalytic sector F_1_ (formed by subunits *α* and *β*, three copies each) and membrane intrinsic proton-conducting sector F_o_ (made of subunits c, which in mammals forms an 8-member ring, a, and supernumerary subunits e, f, g, A6L, DAPIT, and 6.8PL) [36, 37]. They are connected through central and peripheral stalks comprised of subunits *γ*, *δ*, and *ε* and subunits b, d, F_6_, and OSCP respectively [36, 38]. Two approaches to manipulate ATP synthase have been employed to test this theory, and two major hypotheses on the role of this complex of proteins in PTP formation have emerged in the last 5 years (discussed below).

#### 2.1.1. Dimer Hypothesis

The first hypothesis proposes that PTP is formed by PTP-specific conformation of dimers of ATP synthase when it shifts from ATP-synthetizing to ATP-dissipating nanomachine, the “dimer” hypothesis. In a seminal work, Giorgio and colleagues [30] have demonstrated that purified bovine ATP synthase dimers, but not monomers, can conduct currents when inserted into planar lipid bilayers which are activated by Ca^2+^ and oxidizing agents. These currents were closed upon addition of ADP/Mg^2+^ (established PTP desensitizers) and correlated well with the currents observed in patch clamped IMM preparations (mitoplasts) attributed to mitochondrial mega channel (MMC), an electrophysiological equivalent of the PTP [39, 40]. These findings were later supported by similar studies in yeast and Drosophila [31–33]. Interestingly, even though channel activities of ATP synthase dimers were observed in all species tested and possessed the same regulatory pattern (i.e., were activated by Ca^2+^ plus thiol oxidants and inactivated by ADP/Mg^2+^), their maximum conductance state differed, varying from 1 ns to 300 ps to 53 ps in *B. taurus*, *S. cerevisiae*, and *D. melanogaster*, respectively [30–32]. These results imply that the pore diameter may vary between species.

The “dimer” hypothesis was further endorsed by studies of yeast PTP in strains lacking ATP synthase dimerization subunits e and g. These mitochondria could handle about twice as much Ca^2+^ compared to wild-type preparations before the yeast PTP occurred [31] and do not swell in sucrose-based media upon PTP opening [33]. Further, Carraro et al. have demonstrated that in contrast to what was previously believed, dimers are still present in mutant mitochondria. They are less stable, however, and require crosslinking with Cu^2+^ to be detectable by BN-PAGE [33]; thus, they were missed in previous studies [31]. In support of these findings, the cryo-EM structure of yeast ATP synthase dimer revealed that subunits e and g do not directly participate in dimer formation, but rather facilitate it through contacts with subunits b and f [41]. Interestingly, when put in planar lipid bilayers, Cu^2+^-stabilized dimers devoid of subunits e and g conducted Ca^2+^-dependent channel activity, yet with 10-fold lower currents than controls. These currents could be further reduced by eliminating the first transmembrane domain of subunit b [33]. Thus, the full conductance channels might reflect the long-lasting PTP openings that result in mitochondria demise, while the low conductance channels observed above could well reflect the flickering state of the PTP with distinct physiological roles, e.g., a quick Ca^2+^ release as observed in Drosophila [32].

#### 2.1.2. c-Ring Hypothesis

An alternative or “c-ring” hypothesis has been the subject of much controversy. This hypothesis is primarily advocated by Bonora's group [35, 42, 43] and supported by findings gathered by Alavian et al. [44] and Azarashvili et al. [45]. It proposes that the central component of the PTP is constituted by the c subunit of the ATP synthase. In HeLa cells, interference with c subunit expression levels has resulted in a change in PTP opening probability. While downregulation caused its desensitization, overexpression resulted in sensitization [43, 44]. Moreover, when purified c subunits were added to isolated mitochondria, excess c subunit induced the PTP, and when added to bilayer membranes, conducted currents that were cation selective [45] rather than showing lack of selectivity normally associated with PTP function [46, 47]. An additional study [44] detected PTP resembling currents upon c subunit incorporation into proteoliposomes. In support of c-ring being an important player in PTP formation, Morciano et al. discovered 1,3,8-triazaspiro[4.5]decane derivatives as ligands of c subunit. These molecules delayed PTP opening upon Ca^2+^ overload and were protective in *ex vivo* models of ischemia-reperfusion injury [48].

Mechanistically, supporters of this model propose that, upon excess Ca^2+^ and ROS, dissociation (rather than association as suggested in “dimer” hypothesis) of dimers and detachment of F_1_ sector would allow for conformational change of c-ring which would then form the PTP [42]. The major issue with “c-ring” hypothesis is that significant structural alterations have to occur upon relatively modest change in the surrounding environment; they need to be quick and reversible. First, F_1_ sector would need to be removed from F_o_, and second, the lumen of c-ring would have to be emptied of lipids to allow the passage of molecules upon PTP opening. Neither is an easy task. Indeed, it was experimentally determined that as much as 2M urea is required to dissociate F_1_ from F_o_ and it is hard to believe comparable conditions would form in the mitochondrial matrix [18]. Moreover, Zhou et al. have performed atomistic simulations of c-ring from two species, *S. cerevisiae* and *B. pseudofirmus*. Their results concluded that hydration of the lumen of the c-ring pore, required to allow the conducing state to form, is highly unlikely [49]. Regardless, even if hydrated under certain circumstances, the channel would not be only anion selective but also the predicted conductance values (2.5 ps for K^+^ and 116 ps for Cl^−^) would be inconsistent with properties of the PTP [49].

Lastly, Walker laboratory disrupted all three genes encoding for c subunit in order to determine if it is involved in PTP formation. He et al. [50] reported that, despite the loss of c-ring (alongside with a and DAPIT), mutant cells still displayed a CsA-sensitive Ca^2+^-induced Ca^2+^ release and membrane depolarization typically associated with the PTP that was comparable to parent cells. However, Neginskaya et al., while testing the same cells, found that mutants lacking c subunit are more sensitive to Ca^2+^-triggered membrane depolarization and thus, by inference, PTP opening [51]. Further, patch clamp analysis failed to register typical to PTP-conducing channels (~1.5 nS) in mutant preparations, yet observed the emergence of much smaller, 300 pS channels [51], putting subunit c back in contention as critical component of the PTP, be it direct or indirect.

#### 2.1.3. Additional Studies

In tests of ATP synthase as a fundamental component of the PTP, Walker and Bernardi's laboratories have taken two distinct approaches. Walker's group generated HAP1 clonal cell lines lacking select ATP synthase subunits and has established that subunits c, b, OSCP, a, and A6L (as set by Masgras et al. [52]) are dispensable for pore formation [50, 53]. It is important to note that in cells devoid of subunits c, b, and OSCP, ATP synthase fails to assemble properly [50, 53]. Regardless, all of the lines created showed comparable sensitivity to Ca^2+^-induced PTP activity (including Ca^2+^ release and mitochondrial membrane depolarization) to wild-type controls and sensitivity to CsA. The additional finding of one of these studies was that PTP activity was desensitized by CsA in OSCP-null line [53], a rather unexpected observation refuting the previous findings [30] of OSCP subunit being the PTP-modulating binding partner of the mitochondrial peptidyl-prolyl *cis-trans* isomerase D (cyclophilin D, CyPD), thus introducing yet another mystery into PTP complex. However, despite the fact that the “pore” of the PTP must form in the membrane, the essential role of membrane subunits e, g, f, and 6.8PL in mammalian PTP formation has yet to be established.

Bernardi and colleagues, on the other hand, sought to determine the sites of action of major PTP regulators, namely, Ca^2+^ and pH, by site-directed mutagenesis of individual proteins within the ATP synthase complex. They have found that Ca^2+^ binding to catalytic site of the *β* subunit, possibly by replacing Mg^2+^, would cause local conformational change which would propagate through OSCP subunit and lateral stalk of ATP synthase to the IMM to form a pore within transmembrane subunits of the enzyme. The T163S mutation in subunit *β* rendered HeLa cell mitochondria less sensitive to Ca^2+^-triggered PTP opening and to cell death, as well as decreased the number of apoptotic nuclei in zebrafish embryos [54]. In subsequent work, this group identified a unique histidine of the OSCP (H112) as responsible for the PTP inhibition at acidic pH. Hek293 cells in which this His was changed to Gln or Tyr were refractory to (i) MMC inhibition and (ii) prevention of Ca^2+^ overload-induced mitochondrial swelling upon pH switch from 7.4 to 6.5. Further, mutant cells failed to be protected from cell death at acidic pH upon anoxic conditions [55]. Molecular dynamic simulations suggested that protonation of H112, similarly to what was modelled for subunit *β* T163S mutant [54], would cause a conformational change in enzyme's lateral stalk which would then prevent channel formation. Finally, the elegant study led by Guo et al. [56] identified an evolutionarily conserved residue within yeast subunit g (R107) as a target of phenylglyoxal, an established PTP modifier [57, 58].

### 2.2. ANT: Reemergence of an Old Player

While studying the MMC properties in mitochondria lacking c subunit, Neginskaya et al. observed that the detected lower conductance channel was inhibited by CsA, ADP, and bongkrekic acid [51]. Latter features closely resemble channel activities previously reported for purified ANT [59].

The ANT is an integral IMM protein of solute carrier family which facilities ADP exchange for ATP between cytosol and mitochondrial matrix. It was included in the early models of the PTP for several reasons. First, it was found that its ligands, atractylate and bongkrekate, modulate the PTP. The former favors PTP opening and the latter favors PTP closing in the presence of Ca^2+^ [60, 61]. Second, ANT copurified with VDAC and hexokinase in detergent membrane extracts and displayed properties resembling those of PTP after reconstitution in liposomes alone or in concert with VDAC and CyPD [59, 62, 63]. Later studies determined that mitochondria from the ANT1/2 null mouse livers, although losing a detectable response to atractylate, bongkrekate, and ADP and being less sensitive to Ca^2+^-induced Ca^2+^ release, still underwent a CsA-sensitive PTP. These studies led to conclusion that ANT is not essential for PTP formation [28]. However, this early study missed the third isoform, the ANT4. ANT4 is predominantly expressed in the testis, but, at least in the mouse livers, steps in once ANT1/2 are ablated [64, 65]. Mitochondria lacking all three isoforms showed striking resistance to Ca^2+^-induced PTP opening and greatly reduced channel conductance in patch clamped mitoplast. The detected currents were not inhibited by ADP/Mg^2+^ [65]. Moreover, recently, ANT1 was identified as a likely voltage sensor of the PTP. Study led by Doczi [66] confirmed that the absence of ANT1 results in delayed PTP opening in response to Ca^2+^ overload and treatment with H_2_O_2_ in patient-derived fibroblasts, as well as cultured cells, and demonstrated that cells lacking ANT1 require higher voltage threshold for Ca^2+^-induced PTP activation [66].

Taken together, great progress has been made in recent years in attempt to elucidate the long-sought PTP. A wealth of findings supports the notion of ATP synthase as being a PTP-forming entity [34], yet does not conclusively provide the site and mechanism of pore formation, and it appears that ANT [65, 66] plays a more important role in this phenomenon than initially thought.

## 3. Evidence of PTP Involvement in Mitochondrial Disorders

Compromised PTP activation is recognized to play a pivotal role in vast variety of human disorders [18, 21]. The most studied pathologies that include ischemia-reperfusion injury of different organs, muscular dystrophies, and central nervous system diseases are reviewed in [18, 20, 67]. One of the outcomes of prolonged PTP opening is the reversal of ATP synthase to function as ATP hydrolyzing rather than synthetizing enzyme in an attempt to maintain the IMM potential. In this scenario, not only OXPHOS-derived ATP is lost, but ATP derived from glycolysis and mitochondrial substrate-level phosphorylation is also consumed [17, 68, 69] which eventually results in bioenergetic crisis. Importantly, compromised energy metabolism is the central dogma of mitochondrial diseases [70]. Below we overview the evidence linking the PTP and mitochondrial disorders in four disease paradigms.

### 3.1. Leber's Hereditary Optic Neuropathy

Leber's hereditary optic neuropathy (LHON) is the most common mtDNA disorder and is most often, but not limited, to mutations in genes encoding components of complex I; additional genes include *MT-ATP6* [71–74] which encodes ATP synthase subunit a. LHON is characterized by (sub)acute loss of central vision which may affect both eyes simultaneously or start in one and then, within several weeks or months, affect the other. The loss of vision is due to degeneration of retinal ganglion cells and the optic nerve [75, 76]. The use of cell lines containing nuclear DNA from one cell and mtDNA from the other (cybrids) proved to be crucial in characterizing mtDNA-related phenotypes. Studies in cybrid cell lines harboring mutations known to cause LHON revealed that these cells (i) are sensitized to Ca^2+^- or oxidative stress-triggered membrane depolarization and cell death compared to controls [75, 77] and (ii) their mitochondria depolarize if challenged with respiratory chain or ATP synthase inhibitors in a ROS- and Ca^2+^-dependent manner [78], effects that could be counteracted by CsA treatment. The ability of CsA to defer cell death prompted a trial of its oral version (Neoral, Novartis) in LHON patients with acute, strictly unilateral optic neuropathy aimed at preventing the involvement of a second eye. The primary end point of this study—the preservation of visual acuity of the second eye—was not achieved [79]; thus, the trial is considered as failed. Yet, it did delay the involvement of the second eye to the median interval of 28 weeks compared to 6-8 weeks reported in the literature [79, 80]. Several reasons for the lack of prevention of disease progression could be put forward. First, CsA might not have been the best drug to test. Due to its immunosuppressive abilities, the drug has to be administered at well-controlled doses. Importantly, the presence of the drug in blood does not mean that it reaches mitochondria in retinal ganglion cells at therapeutic concentrations. The drug has to cross the blood-ocular barrier first, and studies in rats and rabbits did not detect CsA in the ocular tissues upon oral or intravenous administration [81, 82]; similar studies in humans revealed detectable CsA levels in aqueous humor only upon severe uveitis [83]. Moreover, CsA (as discussed below) and all the molecules acting on CyPD only desensitize the PTP, but do not block it. Second, it might be that in order to reach the maximum protective effect, treatment must be started before the pathological process had set in. It is worth noting that 4 out of 5 patients of this study presented with subtle abnormalities of the central visual field of the second eye indicating that degenerative process might have already started [79].

### 3.2. Neurogenic Muscle Weakness, Ataxia, and Retinitis Pigmentosa (NARP)

NARP syndrome was first documented in 1990 [84] and most commonly is due to a point mutation m.8993T>G in mtDNA (when mutational load is between 70 and 90%) which falls into *MT-ATP6* gene encoding ATP synthase subunit a [9]. This mutation results in biochemical traits such as impaired OXPHOS, increase in ROS, and higher mitochondrial potential [8, 9, 85–87]. Studies in m.8993T>G osteosarcoma 143B cybrids revealed that NARP cells were more sensitive to PTP opening and cell death upon ROS, Ca^2+^, lipid, bongkrekic acid, and amyloid *β* challenge which was largely attenuated upon antioxidant treatment.

### 3.3. French-Canadian Variant of Leigh Syndrome

An interesting case with contradicting data is presented by studies of mitochondrial function in fibroblasts from patients harboring a mutation in [88] and the mouse livers lacking [89] the leucine-rich pentatricopeptide repeat containing (LRPPRC) protein. *LRPPRC* gene encodes a protein that stabilizes mitochondrially encoded mRNAs; its absence results in severe defects in the assembly of OXPHOS complexes IV and V [89–92]. Mutations in *LRPPRC* cause the French-Canadian variant of Leigh syndrome, a disease characterized with a sudden metabolic acidosis which often results in early death [93]. Fibroblasts from affected patients presented with mitochondrial network fragmentation, impaired oxidative phosphorylation capacity, lower membrane potential, and increased sensitivity to Ca^2+^-induced PTP opening [88]. The opposite effect in terms of PTP opening was detected in mitochondria coming from the mouse livers lacking LRPPRC. In this model, loss of LRPPRC resulted in multifaceted bioenergetic phenotype, which includes aberrant mitochondrial ultrastructure, OXPHOS defects, reduction in *MT-ATP6* transcript, and impairment of ATP synthase assembly. Yet, these mitochondria were less sensitive to Ca^2+^-triggered PTP opening and almost refractory to CsA treatment despite expressing twice as much CyPD compared to controls [89]. While the first finding enforces the notion that ATP synthase dimers function as PTP-forming units, the latter is quite unexpected because CyPD is viewed as PTP sensitizer and treatment with CsA displaces it from its binding site releasing PTP sensitization. The most plausible explanation for higher CyPD levels causing resistance to Ca^2+^ overload rather than sensitization is that the PTP-regulating CyPD binding site (probably on ATP synthase) has been lost in this model.

### 3.4. Mitochondrial Encephalomyopathy, Lactic Acidosis, and Stroke-Like Episodes (MELAS)

MELAS, first reported in 1984 [94], is a condition that affects many organs, most severely the muscle and the brain. It can be caused by point mutations in a number of genes, the most common being m.3243A>G in *MT-TL1* encoding mitochondrial transfer RNA [95]. As it is true for all mitochondrial disorders, the disease is rare, its pathogenesis is not fully understood, and disease management is largely supportive [96]. Mitochondria in MELAS fibroblasts show elevated resting Ca^2+^, decreased membrane potential, and higher ROS, are swollen with disintegrated IMM, and tend to accumulate around nucleus [15, 77, 97–99]. Studies in cybrids harboring m.3243A>G mutation determined that mutant cells are more sensitive to H_2_O_2_-induced cell death which could be deferred by Ca^2+^ depletion or CsA treatment [77]. Involvement of PTP in this pathology was further supported by findings of Cotán et al. This group reported that cultured fibroblasts from MELAS patients contain mitochondria with reduced membrane potential, increased oxidative stress, and sensitized PTP opening [99].

## 4. Pharmacological Targeting of PTP

In spite of the importance of the PTP in a variety of disease paradigms, its potential as a drug target is currently not fully exploited, in part due to yet unresolved its molecular identity as discussed above. An extensive list of drugs has been reported to delay PTP opening in *in vitro* studies. However, the majority of these drugs have limited efficacy, are active only at high doses and/or affect PTP indirectly, and thus, are of little relevance for clinical use (see [100] for extensive list and commentary). Only CsA, an 11-amino acid cyclic peptide, best known for its immunosuppressive activities due to binding to cyclophilin A, and its nonimmunosuppressive derivatives Debio-025 (Alisporivir) and NIM811, proved to be useful PTP inhibitors. Although these compounds played a crucial role in establishing PTP as a major player in numerous pathologies [18, 101–109], they desensitize the PTP (by displacing CyPD from its site of action) rather than affect the pore itself and thus afford limited efficacy [110].

Recently, significant efforts were put to fill this void. They can be divided into two classes: (i) phenotypic screens for small molecules affecting PTP directly and not acting on CyPD and (ii) rational drug design-based search for novel small molecule CyPD inhibitors.

### 4.1. High-Throughput Screens for CyPD-Independent PTP Inhibitors

A number of groups aimed to discover novel, CyPD-independent small molecule inhibitors of the PTP with the prospect of targeting the channel directly. It would allow the drug-treated mitochondria to resist to higher stress stimuli than could be achieved by targeting CyPD. In all three screening campaigns (as detailed in [110]), the osmotic swelling of isolated liver mitochondria upon Ca^2+^ overload was followed in the presence of test compounds. In two of the studies, the initial hits were then improved through extensive medicinal chemistry optimization strategies and subsequently tested against a variety of PTP-inducing stimuli.

Fancelli et al. tested commercial library of FDA-approved drugs and identified cinnamic anilides as their most potent series. The best of these compounds not only delayed PTP opening *in vitro* in response to Ca^2+^ overload and oxidative stress [111] but also were protective *in vivo*. For example, compound 22 reduced infarct size in a rabbit model of acute myocardial infarction, yet no significant improvement over CsA treatment was observed [111]. In a subsequent study, this group established that treatment with another member of these series (GNX-4728) increased the lifespan of G37R-hSOD1 mice, a murine model of amyotrophic lateral sclerosis, by nearly 2-fold. Moreover, the treatment prevented motor neuron and mitochondrial degeneration, attenuated spinal cord inflammation, and preserved neuromuscular junction innervation in the diaphragm [112].

Using similar strategy, Roy et al. screened the NIH Molecular Libraries Small Molecule Repository collection consisting of ~360,000 molecules [100, 113]. Among validated hits, compounds from the isoxazole [113] and benzamide [114] chemotypes proved to be most promising. Original library compounds exhibited PTP inhibitory activity comparable to CsA (~200 nM) in isolated mouse liver mitochondria and were effective in permeabilized mouse and human cell lines. Importantly, upon several medicinal chemistry optimization rounds, a number of isoxazole analogs effective at low pM (or 10,000-fold lower that CsA) were developed. These compounds prevented PTP opening triggered by Ca^2+^ and by oxidative stress, did not interfere with ATP synthesis or hydrolysis, and were not toxic at effective concentrations [113]. In addition, they demonstrated that isoxazole 60 is beneficial in a zebrafish model of Ullrich congenital muscular dystrophy. Phenotypes of these fish closely resembled clinical traits of human disease [109, 115] by displaying severe myopathy, motor deficits, and ultrastructural defects; all phenotypes were greatly improved upon addition of isoxazole 60 to fish water [113].

Finally, Briston et al. [116] performed a screen on cryopreserved rat liver mitochondria which retained their functionality upon thawing. After testing 50,000 compounds, the group identified ER-000444793 as their most potent molecule. Although this compound delayed PTP opening upon Ca^2+^ overload in both rat and human mitochondria, it was about 2-fold less effective than CsA. It is worth noticing that, in contrast to other studies, no medicinal chemistry optimization has yet been reported.

### 4.2. Rational Drug Design Studies in Search for Nonpeptidic CyPD Inhibitors

To date, a CsA molecular target in mammalian mitochondria, the CyPD, is the only universally agreed upon component of the PTP complex. Yet, it plays a regulatory role rather than forming the pore itself; mitochondria from mice lacking CyPD still undergo the PTP, although require higher Ca^2+^ and oxidative stress loads [117, 118]. Nonetheless, physiological effects that are sensitive to CsA are routinely defined as being due primarily to the activity of the PTP despite the fact that the majority of the 20 cyclophilins encoded by the mammalian genome show some sensitivity to CsA [119, 120]. As a therapeutic agent, CsA shows unfavorable drug-like characteristics, such as high molecular weight, limited solubility, poor bioavailability [121], and a low blood-brain barrier permeability [122]. The latter is of extreme importance as it renders CsA family drugs unsuitable for treatment of neurological disorders. To address these limitations, several groups took rational design approach to look for novel drugs.

In 2005, Guo et al. [123] reported the discovery of novel quinoxaline derivatives; the best (GW5) showed selectivity for CyPD over cyclophilin A, yet it was effective in preventing Ca^2+^-induced mitochondrial swelling and Ca^2+^ release only at high *μ*M range. No further developments have been since reported. Subsequently, Valasani et al. [124] used virtual screening, molecular docking, and CyPD-pharmacophore studies to design and synthetize pyrimidine-based ligands. Yet, the ability of these compounds to inhibit CyPD and the PTP in biological systems awaits verification. Shore at al. built on previous work of Guichou and colleagues [125] and synthetized a group of urea-based small molecule inhibitors of cyclophilins [121]. The most promising compound (19) bound to CyPD with *K*_d_ of 0.4 *μ*M, delayed pancreatitis toxin-triggered mitochondria depolarization and inhibited subsequent necrotic cell death in freshly isolated pancreatic acinar cells. Lastly, Roh's group has recently reported several distinct classes of urea derivatives which exhibited plausible binding with CyPD according to *in silico* molecular docking studies [126–129]. In these studies, compounds were protective against A*β*-induced mitochondrial depolarization and cytotoxicity while they did not affect ATP levels or cell viability *per se*. Yet, the concentrations used (5 *μ*M) are rather high and no experimental evidence was provided that these compounds inhibit CyPD or exert their actions directly through the PTP. Summing up, while plausible efforts were put to increase the selection of CyPD inhibitors and thereby augment their efficacy in modulating PTP opening, further studies are needed to increase their potency, address the selectivity towards CyPD, and show efficacy in animal models.

## 5. Conclusions

The molecular entity of the PTP is the matter of active investigation, which favors ATP synthase as a prime suspect, yet recent studies suggest that other proteins, like ANT, might also contribute. Early on, it was recognized that preventing PTP opening by treatment with PTP inhibitors would be beneficial in a wide range of therapeutically challenging human diseases. Thus, significant efforts with some promising results were invested in developing PTP-specific inhibitors that would overcome the major drawbacks of CsA, yet further studies are needed to advance them from research tools to therapeutics.

A consequence of PTP opening is the disruption of mitochondrial ultrastructure, dissipation of mitochondrial membrane potential, cessation of ATP synthesis, and cell death as a consequence of resulting bioenergetic crisis—a primary characteristic of mitochondrial disorders, a diverse group of diseases with no available treatment yet. Several studies have demonstrated that cells harboring pathogenic mutations are more sensitive to Ca^2+^ challenge and oxidative stress, effects that could be counteracted by CsA treatment and, potentially, by novel small molecules directly targeting the PTP. These data suggest that PTP might be a viable option as a drug target and thus should be further explored to establish a definite link.
